# Evidence for histidine-rich protein 2 immune complex formation in symptomatic patients in Southern Zambia

**DOI:** 10.1186/s12936-018-2400-8

**Published:** 2018-07-09

**Authors:** Christine F. Markwalter, Lwiindi Mudenda, Mindy Leelawong, Danielle W. Kimmel, Armin Nourani, Saidon Mbambara, Philip E. Thuma, David W. Wright

**Affiliations:** 1 Department of Chemistry, Vanderbilt University, Nashville, TN 37235 USA; 2 Macha Research Trust, Choma, Zambia; 3 Present Address: Rusangu University, Monze, Zambia

**Keywords:** *Plasmodium falciparum*, Histidine-rich protein 2, HRP2, Malaria diagnosis, Antibodies, Immune complexes

## Abstract

**Background:**

Rapid diagnostic tests based on histidine-rich protein 2 (HRP2) detection are the primary tools used to detect *Plasmodium falciparum* malaria infections. Recent conflicting reports call into question whether α-HRP2 antibodies are present in human host circulation and if resulting immune complexes could interfere with HRP2 detection on malaria RDTs. This study sought to determine the prevalence of immune-complexed HRP2 in a low-transmission region of Southern Zambia.

**Methods:**

An ELISA was used to quantify HRP2 in patient sample DBS extracts before and after heat-based immune complex dissociation. A pull-down assay reliant on proteins A, G, and L was developed and applied for IgG and IgM capture and subsequent immunoprecipitation of any HRP2 present in immune complexed form. A total of 104 patient samples were evaluated using both methods.

**Results:**

Immune-complexed HRP2 was detectable in 17% (18/104) of all samples evaluated and 70% (16/23) of HRP2-positive samples. A majority of the patients with samples containing immune-complexed HRP2 had *P. falciparum* infections (11/18) and were also positive for free HRP2 (16/18). For 72% (13/18) of patients with immune-complexed HRP2, less than 10% of the total HRP2 present was in immune-complexed form. For the remaining samples, a large proportion (≥ 20%) of total HRP2 was complexed with α-HRP2 antibodies.

**Conclusions:**

Endogenous α-HRP2 antibodies form immune complexes with HRP2 in the symptomatic patient population of a low-transmission area in rural Southern Zambia. For the majority of patients, the percentage of HRP2 in immune complexes is low and does not affect HRP2-based malaria diagnosis. However, for some patients, a significant portion of the total HRP2 was in immune-complexed form. Future studies investigating the prevalence and proportion of immune-complexed HRP2 in asymptomatic individuals with low HRP2 levels will be required to assess whether α-HRP2 antibodies affect HRP2 detection for this portion of the transmission reservoir.

**Electronic supplementary material:**

The online version of this article (10.1186/s12936-018-2400-8) contains supplementary material, which is available to authorized users.

## Background

Antigen-detecting rapid diagnostic tests (RDTs) have been workhorses for detection of malaria at the point of care, accounting for 63% of diagnostic testing of suspected cases in 2016 [1]. In the same year, the World Health Organization estimates that 312 million RDTs were delivered globally [1]. These common tests are most often formatted as lateral flow assays, which use antibodies to capture and detect malarial parasite proteins.

Approximately 75% of the RDTs delivered in 2016 detected only *Plasmodium falciparum*, which is the most prevalent species of human malaria and is responsible for the majority of severe malaria cases and mortality worldwide [1]. Most RDTs specific for this species rely on the detection of *P. falciparum* histidine-rich protein 2 (HRP2), which was the first antigen targeted in commercial tests [2]. HRP2 is a 30 kDa water-soluble protein found in the parasite and host erythrocyte cytoplasm and on the surface of infected red blood cells [3]. The precise function of HRP2 remains unconfirmed, though the primary structure of HRP2 is highly unique; histidine comprises more than 30% of the primary sequence, which consists largely of AHHAHHAAD and AHHAAD repeat motifs [4]. A cleavable sequence at the N-terminus is responsible for export of HRP2, which diffuses into host plasma, allowing for detection in peripheral blood [3, 5]. Clinical concentrations of HRP2 can range from 100 fM to 100 nM, though expression of HRP2 varies over the erythrocytic life cycle of the parasite [6–9]. The unique structure of HRP2 makes it an advantageous biomarker for malaria detection; multiple epitope copies on a single protein result in high-avidity interactions with antibodies present in RDTs, leading to effective capture and detection of HRP2. Thus, it is not surprising that the most sensitive RDTs on the market are based on HRP2 detection, though performance varies significantly by manufacturer [10].

Despite these advantages, there are several drawbacks to using HRP2 as the sole diagnostic marker for *P. falciparum* infections. The biomarker has been shown to persist in circulation up to 52 days beyond successful treatment and parasite clearance [9, 11]. Thus, an HRP2-based test is unable to distinguish between active and recently cleared *P. falciparum* infections. Additionally, HRP2 is not essential to parasite survival, and clinical isolates with *pfhrp2* gene deletions have been observed with increasing frequency around the world [12–18]. Infections lacking *pfhrp2* will result in false-negative results on HRP2-based malaria rapid diagnostics and can threaten elimination efforts.

Another potential source of false-negative RDT results in *P. falciparum*-infected individuals could be the presence of HRP2-specific antibodies (α-HRP2) produced as part of the host immune response to malaria infection. Although there is significant precedent for endogenous antibody interference with immunochromatographic detection of other infectious diseases, including HIV [19, 20], dengue [21, 22], and tuberculosis [23, 24], only a handful of studies have been published on the potential effects of endogenous α-HRP2 antibodies on biomarker detectability [25–28]. In the first of these studies, Biswas et al. measured HRP2 and α-HRP2 in the serum of patients in India with acute *P. falciparum* infections before treatment and over 28 days after treatment [25]. HRP2 decreased gradually over time, with HRP2-specific IgM following the same pattern. Anti-HRP2 IgG titres increased gradually over the 28 days. Importantly, 3 patients who were RDT-negative and microscopy-positive upon enrollment had significantly higher α-HRP2 IgM and IgG titres compared to the 42 RDT-positive individuals, indicating that the presence of these circulating antibodies could interfere with HRP2-specific RDTs [25]. More recently, Ho et al. found endogenous α-HRP2 antibodies were present in the plasma of 25% of symptomatic malaria patients from Cambodia, Nigeria, and the Philippines and 11% of asymptomatic individuals in the Solomon Islands [27]. The group also found that incubating serum from high α-HRP2-titre individuals with in vitro parasite culture resulted in a marked decrease in RDT signal for several RDT brands [27]. Both of these studies suggest that the humoral immune response against HRP2 could decrease the detectability of HRP2, resulting in false-negative RDT readings.

In direct contrast to the aforementioned reports, two investigations have found an absence of endogenous α-HRP2 antibodies in patients from malaria-endemic regions. In a study aimed to determine the immunomodulatory properties of the biomarker, Das et al. found that PBMCs isolated from *P. falciparum*-exposed patients in India did not produce a detectable HRP2-specific antibody response when stimulated with the antigen [26]. Most recently, Taylor et al. evaluated plasma samples from Cameroonian individuals living in a region with high *P. falciparum* transmission with the goal of determining the prevalence, class, subclass, and avidity of circulating α-HRP2 antibodies [28]. Although these patients had robust levels of antibodies specific for other *P. falciparum* antigens, including three malaria merozoite surface proteins (MSP1, MSP2, and MSP3), the levels of detectable circulating α-HRP2 antibodies were no different from those of malaria-naïve control patients from the United States.

These discordant results in the literature led to this investigation of whether individuals living in a low-transmission region in Southern Zambia produce HRP2-specific antibodies that could interfere with HRP2 detection. However, unlike the four published studies, all of which detected freely available circulating α-HRP2 using a direct immunoassay format, this work specifically sought to determine whether patient samples contain HRP2 immune complexes. (Note: in this study, immune complexes refer to antibodies bound to antigen.) To do this, magnetic particles were used to isolate IgG and IgM (free and complexed) from patient sample dried blood spot (DBS) extracts. The captured antibodies were then exposed to denaturing immunoprecipitation conditions in order to release any complexed HRP2, which was subsequently measured by ELISA. Additionally, free HRP2 in untreated and heated DBS extracts was measured to determine whether signal could be enhanced by dissociating any immune complexes present.

## Methods

### Reagents and materials

Human whole blood (CPD) was purchased from Bioreclamation IVT (catalog no. HMWBCPD). Recombinant HRP2 protein (rcHRP2) was generously provided by PATH (Seattle, WA). *Plasmodium falciparum* D6 strain was cultured in-house. Dynabeads^®^ Protein A, Dynabeads^®^ Protein G, and Pierce Protein L Magnetic Particles were purchased from Fisher Scientific (10-002-D, 10-004-D, PI88850). Anti-HRP2 antibodies were purchased from Abcam (ab9203, ab9206, ab30384). TMB One was purchased from Promega (G7431). 903 Protein Saver Cards were purchased from GE Healthcare Life Sciences (10534612). A Fisher Scientific Analog Vortex Mixer (02-215-365) was used for all vortexed incubations. A VWR Digital Dry Heatblock (12621-086) with an external thermocouple (11301-112) was used for sample heating. Absorbance was measured on a Biotek Synergy H4 microplate reader (Vanderbilt University) or Biotek ELx808 microplate reader (Macha Research Trust). All other reagents and materials were purchased from either Fisher Scientific or Sigma Aldrich.

### HRP2 enzyme-linked immunosorbent assay (ELISA)

A previously reported HRP2 ELISA protocol was employed [9, 29]. Briefly, 100 µl of 1 µg/ml α-HRP2 IgM (ab9206, clone PTL3) was added to the wells of an Immulon 2HB 96-well plate for 1 h. After 3 washes with 1× phosphate buffered saline with 0.1% Tween-20 (PBST), the plate was blocked with 300 µl of 5% BSA in PBST for 2 h. Standards and samples (100 µl) in ELISA sample buffer (PBST with 0.1% BSA) were then added to the plate for 2 h. Next, 100 µl of 0.5 µg/ml α-HRP2 conjugated to horseradish peroxidase (HRPx) (ab30384, clone MPFG55P) in PBST with 0.5% BSA was added for 1 h while protected from light. Signal was generated using TMB One solution, and the reaction was stopped with 2 M H_2_SO_4_ after 10 min. Absorbance was measured at 450 nm. For all ELISAs performed in this study, the average LOD was 0.012 ± 0.004 pM rcHRP2. The average intra-assay variability was 3.4% and the inter-assay variability was 22%.

### Dried blood spot (DBS) preparation and extraction

To prepare control mock DBS patient samples, in-house *P. falciparum* D6 culture (stock: 43,600 parasites/µl) and a high affinity α-HRP2 mouse monoclonal antibody (ab9203, clone C1–13) were spiked into whole blood to desired concentrations and spotted (10 µl) onto Protein Saver 903 cards. The DBS were air-dried for a minimum of 4 h and a maximum of overnight. A modified office hole-punch (Office Depot^®^ #825232 with punch tray removed) was used to remove DBS from the cards. Five punches of clean DBS cards were performed between each sample punch to minimize cross-contamination. Each DBS was placed in a 2-ml microcentrifuge tube, and 300 µl of PBST was added to each tube. The tubes were placed on a vortexer at maximum speed (3200 rpm) for 10 min and then a mini-centrifuge for 1 min to remove bubbles. The supernatant was removed and reserved for analysis. For each sample, half of the DBS extract supernatant was added to a separate 2-ml microcentrifuge tube and placed on an 80 °C heat block for 10 min. These heated samples were then allowed to cool to room temperature before ELISA analysis.

### DBS ELISA

ELISA plates were prepared as described above. Heated and untreated DBS extracts were diluted tenfold in ELISA sample buffer (PBST with 0.1% BSA), and 100 µl of each diluted sample was placed on the plate in duplicate. Each plate also contained an rcHRP2 standard curve (0–10 pM) in sample buffer. Incubation times, washes, and addition of detection antibody, HRPx substrate, and quenching solution were identical to the HRP2 ELISA protocol above. Signal was measured at 450 nm.

### HRP2 immune complex pull-down assay

In order to determine the amount of HRP2 complexed with antibodies in each sample, 10 µl of untreated DBS extract was added to 40 µl of PBST in a 1.5-ml microcentrifuge tube. Next, 30 µl of a 1:1:1 mixture (10 mg/ml) of Dynabeads^®^ Protein G, Dynabeads^®^ Protein A, and Pierce™ Protein L magnetic beads was added to the diluted DBS extract, and samples were incubated on a vortexer (3200 rpm) for 10 min. Using a magnetic tube holder (Invitrogen MagnaRack CS15000), the supernatant was removed. Next, the magnetic beads were washed by vortexing with 50 µl of PBST, and the wash supernatant was removed using the magnetic tube holder. To elute any HRP2 complexed to antibodies captured by the beads, a classic denaturing immunoprecipitation protocol was followed: 40 µl of 0.5 M glycine buffer (pH 3) was added to the beads, which were vortexed and then placed on an 80 °C heat block for 10 min. Using the magnetic rack, the supernatant was removed from the beads and placed in a 1.5-ml microcentrifuge tube before the addition of 15 µl 1 M TRIS pH 8. After neutralization, 50 µl of ELISA sample buffer was added to each tube. This process was performed in duplicate for each sample. Thus two 100-µl neutralized and diluted samples were placed on an ELISA plate to measure HRP2 concentrations for each DBS sample. ELISAs were carried out as described above.

### Study setting, patient recruitment, and ethics

Clinical DBS samples were collected in the catchment area of Macha Mission Hospital in Choma District, Southern Province, Zambia, a rural 1200 km^2^ area where roughly 30,000 individuals live [30]. In this region, there is a single rainy season from November through April in which malaria transmission peaks, though the prevalence of malaria has declined steadily over the last decade to less than 1% [31, 32]. Patients were enrolled into the present study from both passive and reactive surveillance settings. For the former, patients presenting to Macha Mission Hospital with fever (> 37.5 °C) were prescribed a malaria RDT (SD Bioline Pf) according to Hospital protocol. After provision of written informed consent and completion of a questionnaire, capillary whole blood was collected by finger prick once the prescribed RDT was performed. In the case of minors under 18 years of age, consent and survey responses were requested from a parent or guardian. As fever was a requirement for recruitment in the clinic, all patients providing samples in this setting were classified as symptomatic. 49 patients were enrolled in the Hospital setting in March and April 2017. In addition to the clinic setting, patients already enrolled in Step D of the reactive screen-and-treat efforts implemented in this area of Southern Province, Zambia were recruited for this study [33]. These patients were either known index malaria cases, living in the same household as an index case, or living in a household located within 140 m of an index case. Patients were recruited for this study only if they or a parent/guardian provided written informed consent. Finger prick capillary blood was collected after the prescribed RDT (SD Bioline Pf) for Step D surveillance was performed. In this reactive surveillance setting, 55 patients were enrolled in March–April 2017. A total of 104 samples were analysed in this study. This study and sample collection were performed under IRB approval (MRT IRB # E.2014.01.v4.0) and after approval for the study was granted by the Zambian National Health Research Authority (MH/101/23/10/1).

### Patient sample DBS preparation and storage

Finger-prick whole blood samples were collected in 300 µl SAFE-T-FILL Capillary Blood Collection Tubes containing sodium citrate (Ram Scientific). Whole blood samples (5 µl) were run on Paracheck Pf RDTs, which were measured using an ESEQuant Lateral Flow Reader (Qiagen) with a cut-off of 30 mV. Next, 10 µl spots were placed on Protein Saver 903 cards and allowed to dry at room temperature overnight. Samples were either punched and analysed the next morning or placed in a zip-lock bag with desiccant and stored at − 80 °C. All samples were analysed in April and May 2017.

### DNA extraction and PCR amplification

DNA was extracted from the dried blood spots using the Chelex method as previously described [34], with minor modifications. The spots were punched out with a 6.35 mm hole punch directly into 1 ml of a 0.1% saponin solution and incubated at room temperature for 10 min. After discarding the supernatant, PBS was used to rinse the spot, and 150 µl of 2% Chelex-100 and 50 µl of water were added. Samples were incubated at 100 °C for 8 min. The tubes were centrifuged, and the supernatant was collected. Samples were stored at − 20 °C. Real-time PCR to detect the *P. falciparum* 18S gene was performed with the previously described Fal-F [35] and Plasmo2-R primers and the Falc 6-carboxyfluorescein (FAM)-labelled probe [36]. The primers were used at a concentration of 200 nM each and the probe at 50 nM in QuantiFast Probe PCR Master Mix (Qiagen). Samples and controls were run in triplicate. A standard curve was included with each 96-well plate. Reactions were amplified and analysed with the Roche Lightcycler 480 II using the following cycling conditions: initial denaturation at 95 °C for 5 min and 45 cycles of 95 °C for 10 s and 60 °C for 30 s.

### Determination of free and immune-complexed HRP2 in patient samples

Two DBS per patient sample were extracted. Half of the DBS supernatant was reserved (“untreated”), and the remainder was heated on an 80 °C heat block for 10 min. 20 µl of untreated DBS extract was used to determine the amount of HRP2 complexed with antibodies using the protein A/G/L extraction protocol described above in duplicate. Additionally, the untreated and heated DBS extracts were diluted tenfold in ELISA sample buffer, and a DBS ELISA was performed in duplicate as detailed above. Samples for which signal did not fall in the linear range were re-run at the appropriate dilutions. Note that in this manuscript, “free” HRP2 refers to HRP2 not bound in immune complexes regardless of whether it was solubilized in the plasma or originated from within infected erythrocytes.

### Data analysis

HRP2 concentrations were interpolated from rcHRP2 standard curves run on each plate. Limits of detection were calculated as the concentration at which the absorbance was equal to *s*_*blank*_ + *3SD*_*blank*_. Intra- and inter-assay variability (%CV) were determined as the average relative standard deviation of duplicate measurements on a single plate and the average relative standard deviations of all measurements at each concentration on the standard curve across all assays performed over the duration of the study, respectively. All error bars represent the standard error of measurement. Where applicable, Spearman correlation coefficients were calculated when the data were found to not be normally distributed according to the D’Agostino and Pearson normality test. A paired *t* test was used to determine whether the measurable [HRP2] was significantly different in untreated and heated samples (α = 0.05). An individual sample was defined as “enhanced” if the concentration of HRP2 in the heated sample was significantly greater than that in the untreated sample (one-sided t-test, α = 0.05). A sample was determined to contain HRP2 complexed to IgG or IgM if the concentration measured in the protein A/G/L extraction protocol was significantly different from the limit of detection of the HRP2 ELISA (Student’s t-test, α = 0.05).

## Results

### Dissociation of pre-formed HRP2 immune complexes

**Fig. 1:**
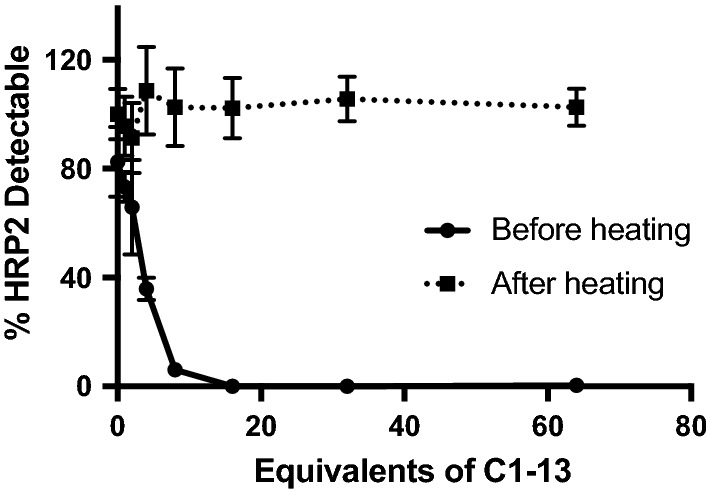
Increasing equivalents of α-HRP2 antibodies reduced free HRP2 detectable by ELISA by forming immune complexes. Heating samples at 80 °C for 20 min dissociated these complexes, completely restoring ELISA signal, regardless of α-HRP2 excess

A series of laboratory controls were performed before analysis of patient samples. First, rcHRP2 was incubated with varying equivalents of a mouse monoclonal antibody (clone C1–13), which was previously shown to have excellent affinity for rcHRP2 [37]. A portion of these HRP2-antibody mixtures was then heated to 80 °C for 20 min, and the detectable HRP2 concentrations in both untreated and heated samples were measured by ELISA. The C1–13 clone was not employed in the ELISA protocol. As shown in Fig. 1, α-HRP2 antibodies interfere with ELISA detection of HRP2 by forming immune complexes. Heating samples at 80 °C dissociates these complexes and completely restores ELISA signal, regardless of α-HRP2 excess. This dissociation was found to be irreversible. Fully restored HRP2 ELISA signal was maintained even after allowing dissociated samples to cool for several hours. This is consistent with the literature; Leow et al. reported the melting temperature of C1–13 as 72 °C, and the rapid rate of heating in this experiment (i.e. placing samples directly on an 80 °C heat block) likely resulted in irreversible precipitation of the denatured IgG [38, 39]. Additional optimization found that 5 min heating time was sufficient to fully dissociate complexes, so a final heating time of 10 min was chosen for further experiments.

### Optimization of HRP2 immune complex pull-down assay

**Fig. 2:**
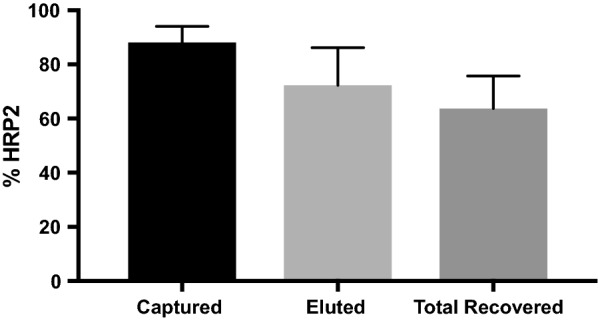
Using the optimized conditions for the immune complex pull-down assay, 90% of complexed HRP2 was captured and 70% of the captured biomarker was eluted, resulting in an overall recovery of about 60%. Note that these results originated from samples in which all detectable HRP2 was initially complexed to C1–13

A pull-down assay was developed to determine the quantity of HRP2 complexed with antibodies in a given sample. Magnetic particles functionalized with Proteins A, G, and L were incubated with diluted DBS extracts in order to capture all IgG and IgM from a sample. These beads were washed with buffer before they were subjected to denaturing immunoprecipitation conditions that released any complexed HRP2. The supernatant was removed and neutralized, and HRP2 was quantified by ELISA. To optimize this process, rcHRP2 (500 pM) and 20 equivalents of C1–13 (10 nM) were spiked into whole blood, incubated for 10 min to allow immune complex formation, and spotted onto DBS cards. It was found that DBS extracts required a minimum fivefold dilution in order to maximize HRP2 capture, and multiple bead mixing techniques (vortexer, orbital microplate shaker, and rotisserie) were found to perform similarly to one another (Additional file 1: Fig. S1). The final optimized system successfully captured 90% of HRP2 in the sample, and the immunoprecipitation protocol released 70% of the captured biomarker. Thus, the pull-down assay successfully detected approximately 60% of HRP2 when all antigen was bound in immune complexes (Fig. 2).

### Evaluation of immune complex dissociation and pull-down assay in mock patient samples

In order to evaluate the optimized immune complex dissociation and pull-down protocols, a panel of mock patient samples was prepared by spiking in-house D6 *P. falciparum* culture and C1–13 antibodies into human whole blood. The panel of mock samples was designed to test the limits of the optimized systems and included the following whole blood controls: (1) no parasites and no C1–13, (2) no parasites with C1–13 (50 nM) (3) varied parasite concentration (up to 6400 parasites/µl) and no C1–13, (4) constant parasite concentration (2000 parasites/µl) with varied C1–13 (up to 100 nM), and (5) varied parasite concentration with constant C1–13 (50 nM). These DBS samples were analysed according to the optimized dissociation and pull-down protocols.

**Fig. 3:**
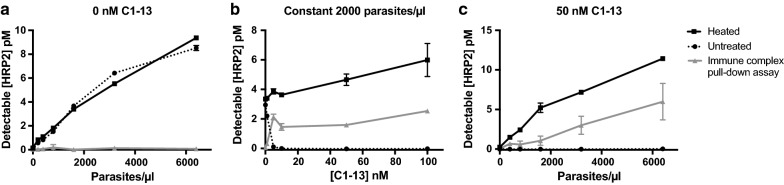
Evaluation of optimized immune complex dissociation protocol and pull-down assay in mock patient samples. **a** In the absence of α-HRP2 antibodies, no HRP2 was detectable in the immune complex pull-down assay. Additionally, heating free HRP2 did not diminish ELISA signal. **b** Increasing equivalents of α-HRP2 C1–13 decreased ELISA signal, but heating fully restored that signal. **c** Immune complex pull-down recovered 60% of complexed HRP2 over a wide range of parasite concentrations

As shown in Fig. 3a, in the absence of C1–13, no HRP2 was detected in the immune complex pull-down assay, even at high parasite densities. Importantly, this demonstrated that only HRP2 complexed to α-HRP2 antibodies, and *not* free HRP2, is pulled down in the bead-based assay, regardless of the magnitude of HRP2 concentration present. The data in Fig. 3a also demonstrated that no signal is lost when samples containing only free HRP2 are subjected to heating. Additionally, the immune complex pull-down assay worked well over a broad range of α-HRP2 C1–13 concentrations, and dissociating complexed samples by heating completely restored positive signal (Fig. 3b). The pull-down assay also demonstrated the expected 60% HRP2 recovery over a broad range of parasite concentrations in the presence of excess C1–13 (Fig. 3c). Taken together, these data show that the optimized protocols behaved as expected for all controls in samples that closely mimicked patient dried blood spot samples.

### Free HRP2 levels in untreated and heated patient DBS extracts

**Fig. 4:**
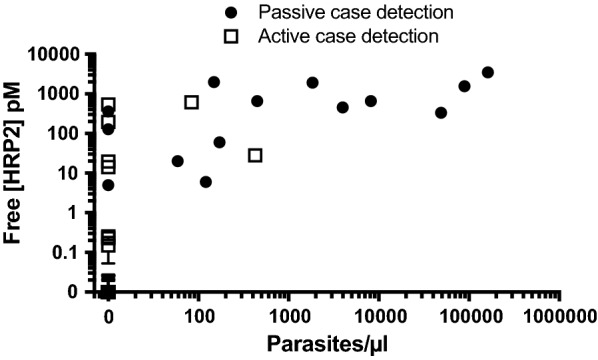
Relationship between free [HRP2] and parasitaemia at the time of sample collection. A significant positive association (Spearman coefficient: 0.7623, P < 0.0001) was found

**Fig. 5:**
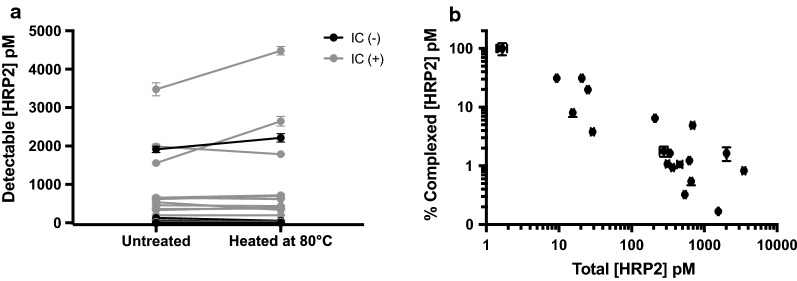
Dissociation and pull-down of HRP2 immune complexes in patient samples from rural Southern Zambia. **a** Heat dissociation of HRP2 immune complexes did not result in a statistically significant overall enhancement effect. Grey color indicates samples in which immune complexed HRP2 was detected using the pull-down assay. **b** Relationship between the % of HRP2 in complexed form and the total HRP2 in patient DBS samples

A total of 104 patient samples were analysed in this study. *P. falciparum* infection status was determined by PCR, and HRP2 was measured by ELISA. As shown in Table 1, 81 (78%) recruited patients had no *P. falciparum* infection and no detectable free HRP2. The remaining 23 samples (22%) were HRP2-positive, 13 of which were parasitaemic according to PCR. All individuals with *P. falciparum* infections had detectable HRP2 levels. Overall, a positive association between parasitaemia and detectable free HRP2 was found (Spearman coefficient: 0.7623, P < 0.0001) (Fig. 4). The results of heating DBS extracts are shown in Fig. 5a. Heating DBS extracts did not result in an overall higher concentration of detectable HRP2 compared to untreated samples (paired t-test, P = 0.1898). On an individual level, a significant increase in detectable HRP2 was observed in 6 patient samples (P < 0.05). All patients for which HRP2 detectability in DBS extracts was significantly enhanced by heating were parasitaemic, and the initial free [HRP2] in the corresponding DBS extracts was greater than 600 pM. Among these 6 samples, the average signal enhancement factor was found to be 1.3 ± 0.2. In other words, in these 6 samples, 30% more HRP2 was detectable in the heated samples compared to the untreated samples.

**Table 1:** Patient samples stratified by collection strategy, *Pf* PCR results, and detectable free HRP2

PCR	Free HRP2	Passive case detection (hospital) (n)	Reactive case detection (field) (n)	Total (n)
−	−	35	46	81
−	+	3	7	10
+	+	11	2	13
Total		49	55	104

### HRP2 present in immune complexes in patient DBS extracts

**Fig. 6:**
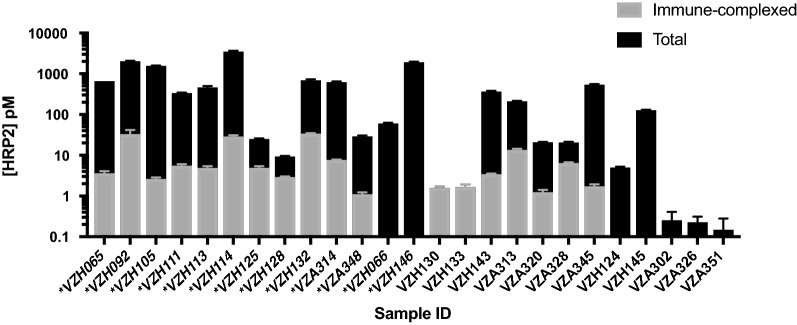
Concentrations of immune-complexed and total HRP2 in all HRP2-positive samples evaluated using the immune-complex pull-down assay. Note that the y-axis is on a logarithmic scale. Sample IDs preceded by asterisks represent patients infected with *P. falciparum* parasites detected by PCR

Immune-complexed HRP2 was detected in the DBS extracts of 18 patient samples, which represents 17% (18/104) of all samples evaluated and 70% (16/23) of samples with detectable free HRP2. As shown in Table 2, 2/18 of the samples containing immune-complexed HRP2 were PCR-negative and did not have detectable free HRP2 (i.e. all HRP2 was in complexed form); 5/18 samples with immune complexes were PCR-negative and had detectable free HRP2 (likely recently resolved infections); and 11/18 were PCR-positive and had detectable free HRP2. Only 6/18 of the samples found to contain immune-complexed HRP2 were collected in the reactive case detection setting. The median percent of total HRP2 present in immune complexes was 2.7% (range 0.17–100%). As visualized in Fig. 6, the percent of total HRP2 present in immune complexes was less than 10% for most patient samples containing HRP2 immune complexes. However, for 5 patient samples, 20% or more of total HRP2 was present in immune-complexed form.

**Table 2:** Patient samples stratified by presence of immune complexed HRP2, Pf PCR results, and detectable free HRP2

PCR	Free HRP2	Immune-complexed HRP2 detected (n)	No immune-complexed HRP2 detected (n)	Total (n)
−	−	2	79	81
−	+	5	5	10
+	+	11	2	13
Total		18	86	104

Immune-complexed HRP2 was not found in 7/23 samples with detectable HRP2 in untreated DBS extracts. The average free [HRP2] in this group was not significantly different from samples in which complexed HRP2 was detected (P = 0.4311). Additionally, there was no significant difference in parasite densities (determined by PCR) between the two groups (P = 0.3072), though most samples without detectable immune-complexed HRP2 were from patients who did not have detectable parasitaemia at the time of sample collection. In the context of the heat dissociation results, free HRP2 signal was significantly enhanced after heating for only 5 (28%) of the samples with detectable immune-complexed HRP2. This discrepancy could arise from the fact that, for many samples with immune-complexed HRP2, the percent of total [HRP2] in complexed form was so low that differences in [HRP2] after dissociation could not be distinguished by duplicate ELISA measurements.

### HRP2 detectability by RDT

The RDT results (Paracheck Pf) for each patient are shown in Additional file 1: Table S1. The overall sensitivity and specificity of the Paracheck Pf RDTs were found to be 78% and 90%, respectively, with the ELISA results for free HRP2 treated as the gold standard. For the group of 18 patients with immune-complexed HRP2, 15/18 patients (83%) were RDT-positive. For the group of patients with free HRP2 and no immune complexes, 3/7 (43%) were RDT-positive. Several of the false-negative results from both groups can be attributed to low (< 1 pM in DBS extract) free HRP2 levels. Importantly, the false-negative RDT rate for patients with immune-complexed HRP2 was not higher than that for patients with no HRP2 immune complexes.

## Discussion

Because HRP2 is so frequently used as a biomarker for *P. falciparum* malaria, it is imperative to thoroughly investigate potential matrix interferants that could result in false-positive or false-negative results in a diagnostic format. Recent conflicting reports call into question whether α-HRP2 antibodies are present in human host circulation and if they could interfere with HRP2 detection on malaria RDTs. Biswas et al. and Ho et al. found circulating α-HRP2 antibodies in patient samples from a variety of endemic areas [25, 27]. In contrast, Taylor et al. found no circulating α-HRP2 in patients from a high-transmission region in Cameroon, and Das et al. found that HRP2 did not stimulate production of α-HRP2 in PBMCs isolated from *P. falciparum*-positive patients from India [26, 28]. Shared among all of these reports is that the presence or absence of freely circulating α-HRP2 was measured in a direct immunoassay format, in which recombinant HRP2 was employed as a capture reagent, and enzyme-conjugated α-human detection antibodies were used to generate signal. There are a couple disadvantages to this approach. First, while the presence of circulating α-HRP2 antibodies suggests the antigen may be present in immune-complexed form, it does not guarantee that this is the case. Immune-complexed antigens could be only transiently present and rapidly cleared by phagocytes. Second, the absence of detectable, freely circulating α-HRP2 does not exclude the possibility that immune-complexed antigen may be present, especially in the case when the antigen concentration is very high. Indeed, Ho et al. found that samples with high HRP2 concentrations generally had lower free circulating α-HRP2 than those with low levels of HRP2 [27].

For these reasons, this work approached the question of endogenous α-HRP2 interference from a new angle and sought to directly interrogate the presence or absence of HRP2-containing immune complexes, rather than freely circulating α-HRP2 antibodies, in patient samples. To this end, two assays were developed and optimized in this work. First, an immune complex dissociation strategy was developed based on the observation that rapidly heating DBS extracts to 80 °C permanently dissociated HRP2 immune complexes. In this assay, the concentration of free HRP2 in DBS extracts was measured before and after heating. For mock samples consisting of DBS spotted with parasitized whole blood, HRP2 ELISA signal was completely diminished when α-HRP2 antibodies were present in excess greater than tenfold; however, heat-based dissociation fully recovered HRP2 ELISA signal. Additionally, heating did not diminish HRP2 signal in the absence of α-HRP2. When this protocol was applied to patient DBS and paired samples were evaluated individually, HRP2 ELISA signal was found to be enhanced after heating for 6 patients, with an average enhancement factor of 1.3 ± 0.2. However, no overall significant difference was found between untreated and heated samples, even for the subset of samples in which immune-complexed HRP2 was found. This could be due to the fact that, in many samples containing complexes, only a small percentage of the total HRP2 was in complexed form, a difference that may not be discernable by duplicate ELISA measurements. Additionally, it is possible that other known interferants, such as rheumatoid factor (RF) and human α-mouse antibodies (HAMA), which can falsely elevate ELISA signal, were denatured as a result of heating, resulting in no net enhancement. Thus, heating samples did not prove to be effective for enhancing overall HRP2 detectability for all samples containing immune-complexed biomarker.

The second developed assay employed protein A, protein G, and protein L-functionalized magnetic particles to isolate IgG and IgM from patient DBS sample extracts. After washing the particles to remove non-specifically bound material, a denaturing immunoprecipitation protocol was applied to release any HRP2 from immune complexes that bound to the particles. The resulting HRP2 signal was measured by ELISA. In mock patient samples, this protocol recovered 60% of immune-complexed HRP2. Importantly, in parasitized whole blood DBS samples, HRP2 was detectable by this method if and only if α-HRP2 antibodies were present. These results indicate that there are no nonspecific interactions between HRP2 and the protein A, G, and L beads in the pull-down assay. It is important to note that, although parasitized whole blood samples spiked with a high affinity α-HRP2 mAb is the closest possible approximation to patient samples, these mock samples are inherently different from patient samples, which may contain antibodies against a multitude of malaria and other antigens.

When the pull-down assay was applied to patient DBS, 18 samples were found to have detectable immune-complexed HRP2, representing 17% of all samples and 70% of samples containing free HRP2. Seven samples containing free HRP2 did not have detectable complexed HRP2. A majority of samples with immune-complexed HRP2 had parasitaemias detectable by PCR (11/18), and nearly all of them (16/18) also contained free HRP2. In the context of previous literature, these results are significant; Ho et al. found that 25% of symptomatic malaria patients had circulating antibodies specific for HRP2 [27]. Although the sample sizes in this study are small, a much larger proportion of symptomatic malaria patients (11/13; 85%) had HRP2 present in immune-complexed form. Additionally, previous studies have suggested that endogenous α-HRP2 antibodies may interfere with HRP2-based malaria diagnosis [25, 27, 40]. However, for nearly three quarters (13/18) of patients with immune-complexed HRP2, less than 10% of the total HRP2 present was in complexed form (Fig. 6). There are three potential explanations for this. First is the possibility that HRP2 concentrations in these samples were vastly greater than the concentration of α-HRP2. Second, HRP2 tagged with endogenous α-HRP2 may be rapidly cleared by phagocytes, thus reducing the relative amount of complexed HRP2 in a sample. The high number of repeated epitopes on HRP2 suggests that this antigen could result in large immune complexes, which rapidly trigger phagocytic clearance [41]. Third, freely circulating α-HRP2 antibodies can only access and bind soluble HRP2, which has been found to represent just a portion of total HRP2 in in vitro studies [3].

Some samples (5) had a large proportion (≥ 20%) of total HRP2 present in immune complexes (Fig. 6). Two samples (VZH130 and VZH133) with detectable immune-complexed HRP2 had no detectable free HRP2, which suggests all HRP2 present in these samples was complexed by α-HRP2. Both of these patients were negative by RDT. In the context of HRP2-based detection by RDT, a high proportion of complexed HRP2 could be worrisome. For example, Scherr et al. found that the visual limit of detection for one brand of malaria RDT was 12.5–100 parasites/µl of in vitro parasite culture, depending on the experience of the reader [42]. This corresponds to 6–50 pM of rcHRP2 used in this study. In this regime, a decrease in detectable HRP2 of 20% or more could be the difference between a reader categorizing a test as positive or negative. However, it is important to note that patients with a high proportion of total HRP2 in complexed form were the exception in this study; most patients had relatively low amounts of complexed HRP2 compared to free HRP2 (median 2.7%), and thus detection by RDT was not affected.

One limitation of this study is that the recovery of the immune complex pull-down assay was found to be 60% in mock patient samples. Thus, the complexed [HRP2] reported herein could underestimate the true complexed HPR2 concentrations. Additionally, most malaria-infected individuals in this study were symptomatic and had enough free HRP2 that detection was not affected by immune complexes. Thus, this study effectively excludes the asymptomatic malaria-infected population, which may have lower free HRP2 levels and is thought to contribute significantly to transmission in some settings [43].

## Conclusions

Overall, this work describes unique methodology for detection of immune-complexed HRP2 and demonstrates its utility in patient DBS samples. The data presented here provide evidence that endogenous α-HRP2 antibodies form immune complexes with HRP2 in the symptomatic patient population of a low-transmission area in rural Southern Zambia. In most patients with immune-complexed HRP2, the proportion of complexed HRP2 was low (< 10%) compared to the total HRP2 present, and HRP2 detection by RDT was not affected. Future studies investigating the prevalence and proportion of immune-complexed HRP2 in asymptomatic individuals will be required to assess whether α-HRP2 affects RDT performance for this portion of the transmission reservoir.

## Additional file

**Additional file 1.** Additional figure and table.

## Abbreviations

HRP2: *Plasmodium falciparum* histidine-rich protein 2
RDT: rapid diagnostic test
DBS: dried blood spot
α-HRP2: anti-HRP2
rcHRP2: recombinant HRP2
ELISA: enzyme-linked immunosorbent assay
PCR: polymerase chain reaction
TMB: tetramethylbenzidine
PBMC: peripheral blood mononuclear cell
