# Supplementary material for: Evidence for histidine-rich protein 2 immune complex formation in symptomatic patients in Southern Zambia
Source: Malar J. 2018 Jul 9;17:256. doi: 10.1186/s12936-018-2400-8 (PMC6038308; doi:10.1186/s12936-018-2400-8)
Supplement: Supplementary file 1 — Additional file 1. Additional figure and table. [file 12936_2018_2400_MOESM1_ESM.docx]

**Evidence for Histidine-Rich Protein 2 Immune Complex Formation in Symptomatic Patients in Southern Zambia**

Christine F. Markwalter,^1^ Lwiindi Mudenda,^1,2^ Mindy Leelawong,^1^ Danielle W. Kimmel,^1^ Armin Nourani,^1^ Saidon Mbambara,^3^ Philip E. Thuma,^3^ David W. Wright^1^*

1 Department of Chemistry, Vanderbilt University, Nashville, TN 37235, USA

2 Present address: Rusangu University, Monze, Zambia

3 Macha Research Trust, Choma, Zambia

* Corresponding Author: david.wright@vanderbilt.edu, +1 615 322 2636

Table of Contents

| Figure S1. Optimization of HRP2 immune complex pull-down assay................................... | S-2 |
| --- | --- |
| Patient demographics.............................................................................................................. | S-3 |
| Table S1. Patient sample data................................................................................................. | S-4 |

**Figure S1**. Optimization of HRP2 immune complex pulldown assay. (A) A minimum DBS extract dilution of 5x is required in order to ensure efficient immune complex capture. (B) Several bead-mixing methods were equivalent in performance. In both experiments, HRP2 and 20 equivalents of C1-13 were spiked into whole blood and incubated for 10 minutes before spotting onto DBS cards.

**Patient demographics**

A total of 104 patient samples were collected for this study. All patients who presented to the hospital (passive case detection) were symptomatic at the time of sample collection. Only two patients recruited through active case detection had *P. falciparum* infections (determined by PCR) and one of these patients reported no symptoms at the time of sample collection. Of the parasite-positive individuals, samples collected from the hospital generally had higher parasite densities (median: 1840, range: 59 – 161764 parasites/µl) than those of individuals recruited through active case detection (median: 255, range 84 – 426 parasites/µl), though no statistical significance was determined due to the low number of parasitemic patients recruited through active case detection.

**Table S1**. HRP2 concentrations measured in the heat dissociation and pull-down assays for all 104 patient samples analyzed in this study.

| **Sample Collection Strategy** | **Sample ID** | **Symptoms?** | **Parasites/**  **µl** | **RDT**  **(+/-)** | **Heat Dissociation ELISA** | | | | **Immune Complex Pull-Down Assay** | | **Total HRP2** | | **% Immune-Complexed HRP2** | |
| --- | --- | --- | --- | --- | --- | --- | --- | --- | --- | --- | --- | --- | --- | --- |
|  |  |  |  |  | **Untreated [HRP2] (pM)** | **Error** | **Heated [HRP2] (pM)** | **Error** | **Complexed [HRP2] (pM)** | **Error** | **pM** | **Error** | **%** | **Error** |
| Passive case detection | VZH064 | Y | 0 | - | 0 | 0 | 0 | 0 | 0 | 0 | 0 | 0 | - | - |
|  | VZH065 | Y | 448 | + | 655 | 35 | 618 | 9 | 3.6 | 0.7 | 658.3 | 35.4 | 0.55 | 0.11 |
|  | VZH066 | Y | 171 | + | 60 | 4 | 14 | 1 | 0 | 0 | 60.2 | 3.8 | 0 | 0 |
|  | VZH067 | Y | 0 | - | 0 | 0 | 0 | 0 | 0 | 0 | 0 | 0 | - | - |
|  | VZH070 | Y | 0 | - | 0 | 0 | 0 | 0 | 0 | 0 | 0 | 0 | - | - |
|  | VZH072 | Y | 0 | - | 0 | 0 | 0 | 0 | 0 | 0 | 0 | 0 | - | - |
|  | VZH073 | Y | 0 | - | 0 | 0 | 0 | 0 | 0 | 0 | 0 | 0 | - | - |
|  | VZH074 | Y | 0 | - | 0 | 0 | 0 | 0 | 0 | 0 | 0 | 0 | - | - |
|  | VZH075 | Y | 0 | - | 0 | 0 | 0 | 0 | 0 | 0 | 0 | 0 | - | - |
|  | VZH076 | Y | 0 | - | 0 | 0 | 0 | 0 | 0 | 0 | 0 | 0 | - | - |
|  | VZH077 | Y | 0 | - | 0 | 0 | 0 | 0 | 0 | 0 | 0 | 0 | - | - |
|  | VZH078 | Y | 0 | - | 0 | 0 | 0 | 0 | 0 | 0 | 0 | 0 | - | - |
|  | VZH080 | Y | 0 | - | 0 | 0 | 0 | 0 | 0 | 0 | 0 | 0 | - | - |
|  | VZH082 | Y | 0 | - | 0 | 0 | 0 | 0 | 0 | 0 | 0 | 0 | - | - |
|  | VZH084 | Y | 0 | - | 0 | 0 | 0 | 0 | 0 | 0 | 0 | 0 | - | - |
|  | VZH090 | Y | 0 | - | 0 | 0 | 0 | 0 | 0 | 0 | 0 | 0 | - | - |
|  | VZH092 | Y | 149 | + | 1990 | 87 | 1787 | 11 | 33 | 12 | 2024 | 88 | 1.6 | 0.6 |
|  | VZH095 | Y | 0 | - | 0 | 0 | 0 | 0 | 0 | 0 | 0 | 0 | - | - |
|  | VZH099 | Y | 0 | - | 0 | 0 | 0 | 0 | 0 | 0 | 0 | 0 | - | - |
|  | VZH100 | Y | 0 | - | 0 | 0 | 0 | 0 | 0 | 0 | 0 | 0 | - | - |
|  | VZH105 | Y | 89236 | + | 1556 | 45 | 2646 | 178 | 2.6 | 0.4 | 1559 | 45 | 0.17 | 0.02 |
|  | VZH108 | Y | 0 | - | 0 | 0 | 0 | 0 | 0 | 0 | 0 | 0 | - | - |
|  | VZH109 | Y | 0 | - | 0 | 0 | 0 | 0 | 0 | 0 | 0 | 0 | - | - |
|  | VZH110 | Y | 0 | - | 0 | 0 | 0 | 0 | 0 | 0 | 0 | 0 | - | - |
|  | VZH111 | Y | 48903 | + | 332 | 11 | 402 | 32 | 5.5 | 0.7 | 338 | 11 | 1.6 | 0.2 |
|  | VZH113 | Y | 3978 | + | 456 | 55 | 436 | 14 | 4.9 | 0.7 | 461 | 55 | 1.1 | 0.2 |
|  | VZH114 | Y | 161764 | + | 3475 | 242 | 4479 | 155 | 29 | 2.6 | 3504 | 242 | 0.83 | 0.09 |
|  | VZH115 | Y | 0 | - | 0 | 0 | 0 | 0 | 0 | 0 | 0 | 0 | - | - |
|  | VZH116 | Y | 0 | - | 0 | 0 | 0 | 0 | 0 | 0 | 0 | 0 | - | - |
|  | VZH121 | Y | 0 | - | 0 | 0 | 0 | 0 | 0 | 0 | 0 | 0 | - | - |
|  | VZH124 | Y | 0 | + | 5 | 0 | 5 | 1 | 0 | 0 | 5.0 | 0.4 | 0 | 0 |
|  | VZH125 | Y | 59 | + | 20 | 1 | 13 | 0 | 5.0 | 0.6 | 25.0 | 1.4 | 20 | 3 |
|  | VZH126 | Y | 0 | - | 0 | 0 | 0 | 0 | 0 | 0 | 0 | 0 | - | - |
|  | VZH127 | Y | 0 | - | 0 | 0 | 0 | 0 | 0 | 0 | 0 | 0 | - | - |
|  | VZH128 | Y | 121 | + | 6 | 0 | 7 | 1 | 2.9 | 0.2 | 9.3 | 0.4 | 31 | 2 |
|  | VZH129 | Y | 0 | - | 0 | 0 | 0 | 0 | 0 | 0 | 0 | 0 | - | - |
|  | VZH130 | Y | 0 | - | 0 | 0 | 0 | 0 | 1.6 | 0.2 | 1.6 | 0.2 | 100 | 16 |
|  | VZH131 | Y | 0 | - | 0 | 0 | 0 | 0 | 0 | 0 | 0 | 0 | - | - |
|  | VZH132 | Y | 8165 | + | 657 | 54 | 718 | 31 | 34.0 | 1.3 | 691 | 54 | 4.9 | 0.4 |
|  | VZH133 | Y | 0 | - | 0 | 0 | 0 | 0 | 1.7 | 0.4 | 1.7 | 0.4 | 100 | 33 |
|  | VZH134 | Y | 0 | - | 0 | 0 | 0 | 0 | 0 | 0 | 0 | 0 | - | - |
|  | VZH135 | Y | 0 | - | 0 | 0 | 0 | 0 | 0 | 0 | 0 | 0 | - | - |
|  | VZH136 | Y | 0 | - | 0 | 0 | 0 | 0 | 0 | 0 | 0 | 0 | - | - |
|  | VZH138 | Y | 0 | - | 0 | 0 | 0 | 0 | 0 | 0 | 0 | 0 | - | - |
|  | VZH139 | Y | 0 | - | 0 | 0 | 0 | 0 | 0 | 0 | 0 | 0 | - | - |
|  | VZH142 | Y | 0 | - | 0 | 0 | 0 | 0 | 0 | 0 | 0 | 0 | - | - |
|  | VZH143 | Y | 0 | - | 362 | 22 | 369 | 17 | 3.4 | 0.2 | 365 | 22 | 0.94 | 0.08 |
|  | VZH145 | Y | 0 | - | 128 | 4 | 52 | 5 | 0 | 0 | 128 | 4 | 0 | 0 |
|  | VZH146 | Y | 1840 | - | 1912 | 115 | 2211 | 155 | 0 | 0 | 1912 | 115 | 0 | - |
| Active Case Detection | VZA301 | N | 0 | + | 0 | 0 | 0.10 | 0.02 | 0 | 0 | 0 | 0 | - | - |
|  | VZA302 | Y | 0 | + | 0.25 | 0.02 | 0.44 | 0.02 | 0 | 0 | 0.3 | 0.2 | 0 | 0 |
|  | VZA304 | N | 0 | + | 0 | 0 | 0 | 0 | 0 | 0 | 0 | 0 | - | - |
|  | VZA305 | N | 0 | + | 0 | 0 | 0 | 0 | 0 | 0 | 0 | 0 | - | - |
|  | VZA306 | N | 0 | + | 0 | 0 | 0 | 0 | 0 | 0 | 0 | 0 | - | - |
|  | VZA307 | N | 0 | - | 0 | 0 | 0 | 0 | 0 | 0 | 0 | 0 | - | - |
|  | VZA308 | N | 0 | - | 0 | 0 | 0.12 | 0.02 | 0 | 0 | 0 | 0 | - | - |
|  | VZA309 | Y | 0 | - | 0 | 0 | 0 | 0 | 0 | 0 | 0 | 0 | - | - |
|  | VZA310 | N | 0 | - | 0 | 0 | 0 | 0 | 0 | 0 | 0 | 0 | - | - |
|  | VZA311 | N | 0 | + | 0 | 0 | 0 | 0 | 0 | 0 | 0 | 0 | - | - |
|  | VZA312 | N | 0 | - | 0 | 0 | 0.14 | 0.07 | 0 | 0 | 0 | 0 | - | - |
|  | VZA313 | N | 0 | + | 196 | 9 | 196 | 12 | 13.6 | 1.3 | 209 | 9 | 6.5 | 0.7 |
|  | VZA314 | Y | 84 | + | 615 | 30 | 697 | 66 | 7.7 | 0.2 | 623 | 30 | 1.23 | 0.07 |
|  | VZA315 | N | 0 | - | 0 | 0 | 0.10 | 0.03 | 0 | 0 | 0 | 0 | - | - |
|  | VZA316 | N | 0 | - | 0 | 0 | 0.08 | 0.04 | 0 | 0 | 0 | 0 | - | - |
|  | VZA317 | N | 0 | - | 0 | 0 | 0 | 0 | 0 | 0 | 0 | 0 | - | - |
|  | VZA318 | N | 0 | - | 0 | 0 | 0 | 0 | 0 | 0 | 0 | 0 | - | - |
|  | VZA319 | Y | 0 | + | 0 | 0 | 0.10 | 0.03 | 0 | 0 | 0 | 0 | - | - |
|  | VZA320 | N | 0 | + | 19.5 | 0.6 | 19.8 | 1.3 | 1.2 | 0.3 | 20.8 | 0.6 | 6.0 | 1.3 |
|  | VZA321 | N | 0 | - | 0 | 0 | 0 | 0 | 0 | 0 | 0 | 0 | - | - |
|  | VZA322 | N | 0 | - | 0 | 0 | 0.08 | 0.03 | 0 | 0 | 0 | 0 | - | - |
|  | VZA323 | Y | 0 | - | 0 | 0 | 0 | 0 | 0 | 0 | 0 | 0 | - | - |
|  | VZA324 | N | 0 | - | 0 | 0 | 0.09 | 0.02 | 0 | 0 | 0 | 0 | - | - |
|  | VZA325 | N | 0 | - | 0 | 0 | 0 | 0 | 0 | 0 | 0 | 0 | - | - |
|  | VZA326 | Y | 0 | - | 0.23 | 0.03 | 0.31 | 0.04 | 0 | 0 | 0.23 | 0.12 | 0 | 0 |
|  | VZA327 | Y | 0 | - | 0.0 | 0.0 | 0 | 0 | 0 | 0 | 0 | 0 | - | - |
|  | VZA328 | Y | 0 | + | 14.2 | 0.9 | 17 | 2 | 6.4 | 0.4 | 20.7 | 1.0 | 31 | 2 |
|  | VZA329 | N | 0 | - | 0 | 0 | 0 | 0 | 0 | 0 | 0 | 0 | - | - |
|  | VZA330 | Y | 0 | - | 0 | 0 | 0 | 0 | 0 | 0 | 0 | 0 | - | - |
|  | VZA331 | Y | 0 | - | 0 | 0 | 0 | 0 | 0 | 0 | 0 | 0 | - | - |
|  | VZA332 | N | 0 | - | 0 | 0 | 0 | 0 | 0 | 0 | 0 | 0 | - | - |
|  | VZA333 | Y | 0 | - | 0 | 0 | 0 | 0 | 0 | 0 | 0 | 0 | - | - |
|  | VZA334 | Y | 0 | - | 0 | 0 | 0 | 0 | 0 | 0 | 0 | 0 | - | - |
|  | VZA335 | Y | 0 | - | 0 | 0 | 0 | 0 | 0 | 0 | 0 | 0 | - | - |
|  | VZA336 | N | 0 | + | 0 | 0 | 0 | 0 | 0 | 0 | 0 | 0 | - | - |
|  | VZA338 | Y | 0 | - | 0 | 0 | 0 | 0 | 0 | 0 | 0 | 0 | - | - |
|  | VZA339 | Y | 0 | + | 0 | 0 | 0 | 0 | 0 | 0 | 0 | 0 | - | - |
|  | VZA340 | Y | 0 | - | 0 | 0 | 0 | 0 | 0 | 0 | 0 | 0 | - | - |
|  | VZA341 | Y | 0 | - | 0 | 0 | 0.10 | 0.04 | 0 | 0 | 0 | 0 | - | - |
|  | VZA342 | N | 0 | - | 0 | 0 | 0 | 0 | 0 | 0 | 0 | 0 | - | - |
|  | VZA343 | Y | 0 | - | 0 | 0 | 0 | 0 | 0 | 0 | 0 | 0 | - | - |
|  | VZA344 | N | 0 | - | 0 | 0 | 0 | 0 | 0 | 0 | 0 | 0 | - | - |
|  | VZA345 | N | 0 | + | 537 | 31 | 336 | 10 | 1.7 | 0.3 | 539 | 31 | 0.32 | 0.05 |
|  | VZA346 | N | 0 | - | 0 | 0 | 0.09 | 0.02 | 0 | 0 | 0 | 0 | - | - |
|  | VZA347 | N | 0 | - | 0 | 0 | 0.11 | 0.03 | 0 | 0 | 0 | 0 | - | - |
|  | VZA348 | N | 426 | + | 28 | 2 | 30 | 2 | 1.1 | 0.2 | 29 | 2 | 3.8 | 0.7 |
|  | VZA349 | N | 0 | - | 0 | 0 | 0 | 0 | 0 | 0 | 0 | 0 | - | - |
|  | VZA350 | N | 0 | - | 0 | 0 | 0 | 0 | 0 | 0 | 0 | 0 | - | - |
|  | VZA351 | Y | 0 | - | 0.15 | 0.14 | 0.11 | 0.03 | 0 | 0 | 0.1 | 0.2 | 0 | 0 |
|  | VZA352 | Y | 0 | - | 0 | 0 | 0 | 0 | 0 | 0 | 0 | 0 | - | - |
|  | VZA353 | N | 0 | - | 0 | 0 | 0 | 0 | 0 | 0 | 0 | 0 | - | - |
|  | VZA354 | N | 0 | - | 0 | 0 | 0 | 0 | 0 | 0 | 0 | 0 | - | - |
|  | VZA355 | N | 0 | - | 0 | 0 | 0 | 0 | 0 | 0 | 0 | 0 | - | - |
|  | VZA356 | N | 0 | - | 0 | 0 | 0 | 0 | 0 | 0 | 0 | 0 | - | - |
|  | VZA357 | N | 0 | - | 0 | 0 | 0.10 | 0.02 | 0 | 0 | 0 | 0 | - | - |
